# Size-Dependent and Property-Independent Passive Microdroplet Sorting by Droplet Transfer on Dot Rails

**DOI:** 10.3390/mi9100513

**Published:** 2018-10-11

**Authors:** Dong Hyun Yoon, Daiki Tanaka, Tetsushi Sekiguchi, Shuichi Shoji

**Affiliations:** 1 Faculty of Science and Engineering, Waseda University, 3-4-1, Okubo, Shinjuku-ku, Tokyo 169-8555, Japan; shojis@waseda.jp; 2 Research Organization for Nano & Life Innovation, Waseda University, 513, Tsurumaki-cho, Waseda, Shinjuku-ku, Tokyo 162-0041, Japan; d.tanaka@aoni.waseda.jp (D.T.); t-sekiguchi@waseda.jp (T.S.)

**Keywords:** microdroplet, sorting, dot rail, transfer

## Abstract

A fully passive microdroplet sorting method is presented in this paper. On the rails with dot patterns, the droplets were sorted in different ways depending on their size. However, the effect of droplet properties on the threshold size of the sorting was eliminated. The droplet positions on two railways and the Laplace pressure of the droplets on the dot patterns allowed selective droplet transfer according to size. Different gaps between the rails altered the threshold size of the transfer. However, the threshold size was independent of the droplet’s surface tension and viscosity because the droplet transfer utilized only the droplet position and Laplace pressure without lateral flow to sort targets. This feature has a high potential for bio/chemical applications requiring categorization of droplet targets consisting of various mixtures as pre- or post-elements.

## 1. Introduction

Microfluidic approaches have recently become an important research method in the fields of chemistry and biochemistry [1,2]. In particular, droplet-based researches provide an efficient volume control, fast reaction, and high throughput screening of thousands of experiments at the miniaturized scale [3,4,5]. Of the numerous kinds of element technologies, target sorting or separation is one of the key technologies in enhancing the efficiency and reliability of the experiments. Specific targets are categorized by optical [6,7], electrical [8,9], thermal [10,11], acoustic [12,13], and pneumatic [14,15] detection or response. 

Size-dependent droplet sorting is an important process throughout the various stages of the experiments. Since the sample volume is directly related to the accuracy of the experiments, the droplet size should be guaranteed before and after treatment or analysis. However, the droplet size is distributed widely from the generation stage depending on flow conditions as well as the environmental conditions such as temperature, humidity, and surface condition of the device [16,17,18,19]. The concentration or uniformity of liquids participating in the droplet generation also affects the sample preparation and analysis [20] Furthermore, large-scale bio/chemical platforms require simultaneous generation and treatment of the targets by integration of various functional elements that influence the internal environments [21,22]. Thus, there is an increasing demand for uniform preparation of droplet-phase samples. 

The passive method requires a simpler system than active sorting methods which involve detection and feedback/response equipment. Thus, this method is more user-friendly and helpful for the integration of other functional elements. 

Passive sample separation or sorting of different volumes has been reported by many studies. Usually, the methods utilize lateral flow in different directions to the main flow. Maenak et al. [23] and Kok et al. [24] employed direct lateral flow by additional injection, and Joensson et al. [25] and Inglis [26] utilized passive migration with lateral flow formed by structural features. Ji et al. [27] and Ding et al. [28] used the structural features mainly for the selective droplet passing such as filtration. Furthermore, based on the balance of inertia and fluidic force, droplet samples migrated to different positions depending on the sample size [29,30,31,32,33,34]. 

The conventional methods have generally focused on sorting or separating the droplets of sufficiently different volumes. The sorting resolutions are highly dependent on the environmental conditions. Furthermore, the flexibility of the materials has caused uncertain results for size-dependent droplet sorting. In our previous works [35,36], we developed a passive droplet sorting method using lateral flow and deformation of the droplets on the functional railway. Using two pair rails, the droplets transferred in different ways depending on their physical conditions. For instance, size, viscosity, and surface tension. 

However, the above method is difficult to sort complex targets, for instance, sorting the specific size when the droplets consist of various materials with different surface tension or viscosity because the properties of droplets affect the sorting results comprehensively. The critical reason for this undesirable result is the large deformation of droplets in the flow field, which is all related to the physical properties.

Hence, this research proposes a method to passively sort droplets at a high resolution according to their size alone. However, deformation of the droplets is fundamentally difficult to avoid when a strong lateral flow or internal flow is involved. Therefore, this research utilizes mainly Laplace pressure instead of flow, which influences the droplets directly. The proposed structure alters the droplet position and boundary depending on its size, and then allows sorting of specific ranges of droplet size.

## 2. Principle 

**Figure 1:**
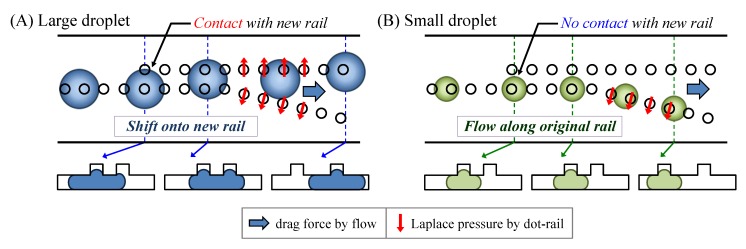
The principle of a size-dependent droplet sorting by selective droplet transfer on dot-rail. (**A**) Transferring of large droplets; (**B**) Guiding of small droplets.

The novel droplet sorting method using size-dependent selective droplet transfer is shown in Figure 1. The method employs a dot-arrayed pattern on Hele-Shaw cell to guide the droplets and transfer them in different ways. When a droplet is injected into a channel with a smaller diameter than that of the droplet, the droplet is sandwiched and flows between the top and bottom walls. The additional structure, dot array, an upper layer on the channel lead the droplet’s position and path because the Laplace pressure squeezes the droplet into a higher structure [37,38,39,40].

As a first step, the dot array guides droplets with a larger size and wider boundary than the diameter of the dot patterns. When the new rail appears downstream and the outer boundary of the droplet reaches new dots, the droplet shifts its position between the two dot rails because of the Laplace pressure. 

When the original rail moves farther away at a constant angle, the large droplets ride on both rails, leaving the original rail because the guiding force of the parallel rail in the flow direction is stronger than that of the angular rail. In contrast, small droplets only ride on the original rail, and the droplets move along the original rail. 

Since this principle does not require strong lateral flow or deformation of the droplets, the effects of the material properties, for instance, flexibility and hardness of the droplet, on the sorting could be negligible. Alternatively, the boundaries of the droplets formed by their size are the dominant factor in determining rail-based droplet transfer. Thus, we can sort the target droplets of specific sizes using the dot arrayed rail structure.

## 3. Device Design and Fabrication 

### 3.1. Computational Analysis 

We performed computational analysis (CFD-ACE+, V2009.0, ESI Group, Paris, France) to evaluate the flow field in the dot array fluidic structure. In particular, the flow distribution is compared to that of the conventional line-based droplet sorting structure [35,36]. The calculation mode was “Flow” and the embedded properties of water were used for the calculation. The dimensions of the channel and the boundary conditions were the same in both cases, whereas the rail patterns differed. Lateral flow in the y-direction in the center of the main channel (height: 15 μm) was visualized and evaluated.

**Figure 2:**
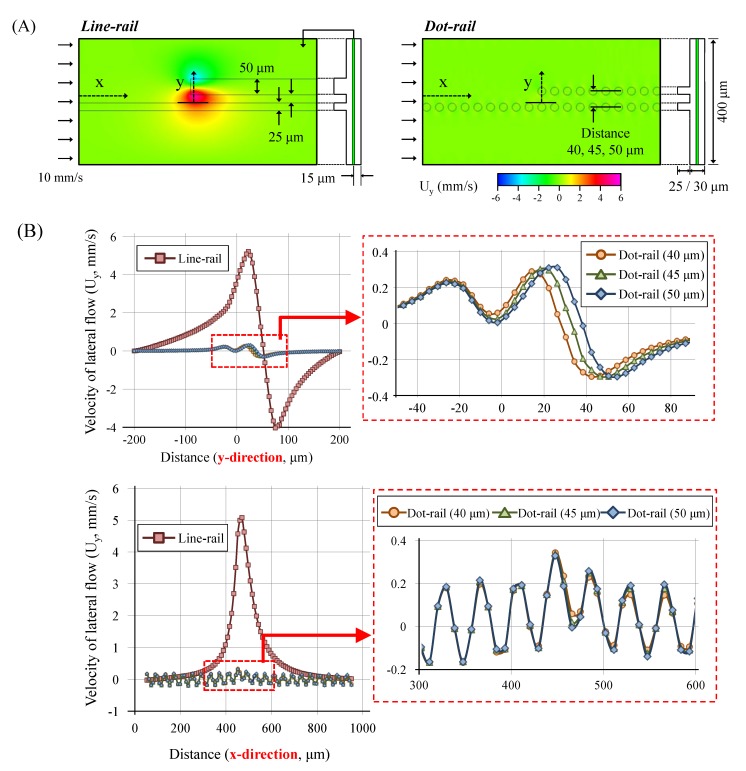
Computation analysis results of the sorting structures. (**A**) Conditions of the analysis and visualization of the flow field. (**B**) Velocity distributions of the side flow in the x- and y-direction at the sorting area.

As shown in Figure 2A, the lateral flow in the dot-rail structure reduced sufficiently compared to that of the line rail. In the case of line-based rail, the lateral flow increased suddenly near the area where the new line started. However, the lateral flow clearly decreased in the same area with the change in rail type to dot rail.

As shown in Figure 2B, the strength of the maximum lateral flow in the dot rail was less than tenth of that in the line rail. Furthermore, the strength variation in the lateral flow according to the change in distance between the dots and the appearance of the new rail were negligible.

Thus, the analytical results for these flow fields and distributions allowed for the prediction that the dot rail structure induces only minimized lateral flow, thereby guiding the droplets without significant deformation.

### 3.2. Device Design and Fabrication 

**Figure 3:**
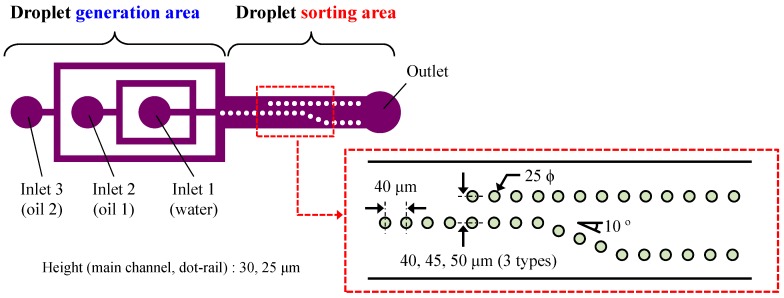
Schematic view of the total device and detailed dimensions of the channel structure.

A schematic view and detailed depiction of the size of the sorting device are shown in Figure 3. The device consists of a droplet generation and a sorting part. A general cross channel was employed for droplet generation and an additional carrier channel was used to control the distance between the generated droplets and their flow speed. The sorting part consisted of a channel layer and a dot-rail layer. The height of the channel layer including droplet generation part is 30 μm and width of the channel is 400 μm. The height of the rail layer is 25 μm and the detailed dimensions of the part are as follows. The diameter of each dot is 25 μm and the distance between the dots is 40 μm. Three distances—40, 45 and 50 μm—between the original and new rail were designed (with gaps of 15, 20 and 25 μm, respectively). Ten dots were overlapped, in which the dots of the original rail were 10° from the new rail. The distance between the two rails downstream was 200 μm.

**Figure 4:**
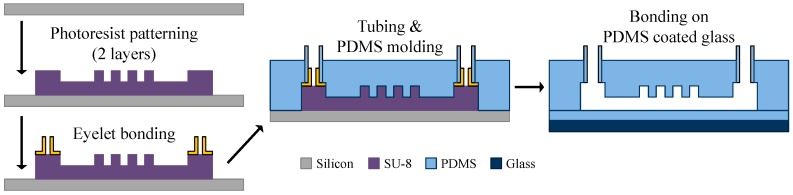
The fabrication process of the sorting device.

The device was fabricated by a photoresist (SU-8, Microchem, MicroChem Corp., Westborough, MA, USA) patterning and polydimethylsiloxane (SILPOT 184, Dow Corning Toray Co., Ltd., Tokyo, Japan) molding process, as shown in Figure 4. The channel and rail layers of the SU-8 mold were formed by two-step lithography on a silicon substrate. Copper eyelets were bonded to the inlet and outlet areas, and then silicone tubes were fitted to the eyelets. The polydimethylsiloxane (PDMS) poured on the mold was cured on a hotplate at 120 °C for 1 h after degassing. The PDMS was bonded with the PDMS coated glass substrate after the oxygen plasma treatment and baked again in an oven at 80 °C for one week to stabilize the surface condition.

### 3.3. Materials and Experimental Setup 

We used water and mineral oil with 5 wt% span80 (37408-32, Kanto Chemical Co., Inc., Tokyo, Japan) as a base material for the droplet and carrier flow. In addition, tween 20 (p1379, SIGMA-Aldrich, St. Louis, MO, USA) and glycerin (075-00616, FUJIFILM Wako Pure Chemical Corp., Osaka, Japan) were employed to modify the surface tension and viscosity of the droplets, respectively.

All of the liquids were injected by syringe pumps (KDS210, KD Scientific, Holliston, MA, USA). The droplet sorting behaviors and results were visualized with a high-speed camera (FASTCAM-NEO, Photron, Tokyo, Japan) and the volumes were calculated by pixel-counting of the droplets downstream without dot patterns. The flow rates of water, oil 1, and oil 2 ranged from 0.001–0.01 μL/min, 0.01–0.05 μL/min, and 0.4–1.0 μL/min, respectively. 

## 4. Results and Discussion

**Figure 5:**
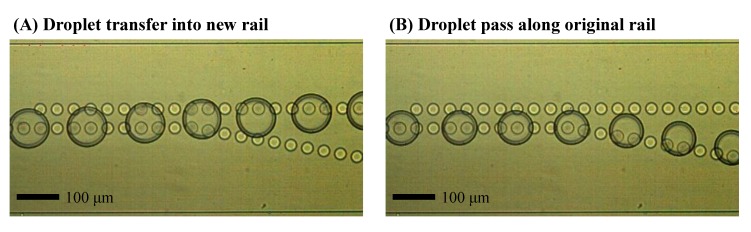
Different droplet routes and transfer behavior in the dot rail according to size and outer boundary. The time interval between each droplet is 0.3 s. (**A**) Transferring of large droplets into the new rail; (**B**) Guiding of small droplets along the original rail.

The behavior of droplet transfer for the size dependent sorting is visualized, as shown in Figure 5 (Supplementary Material, Video S1 and S2, using a rail distance of 45 μm and deionized (DI) water and mineral oil). The generated droplets were simply guided on the center of the dot array (original rail). When the droplet was large, it reached a new dot rail and its position shifted between the two rails as the droplet stretched. The droplet flowed along the rails and then alighted completely from the original to the new rail in the angular rail area. The large droplet transferred the new rail was guided by the new dot array again. 

In contrast, the small droplet that did not reach the new rail flowed along the original rail because the original rail structure only influenced the droplet behavior. It seems that the outer boundary partially entered the dot area. However, the membrane of the droplet did not contact the dot area and the situation was not sufficient to obtain the Laplace pressure for the droplet’s position change. Furthermore, the side flow into the new rail was small and thus remarkable deformation or position shift of the droplet in the area where the new rail starts were not observed.

**Figure 6:**
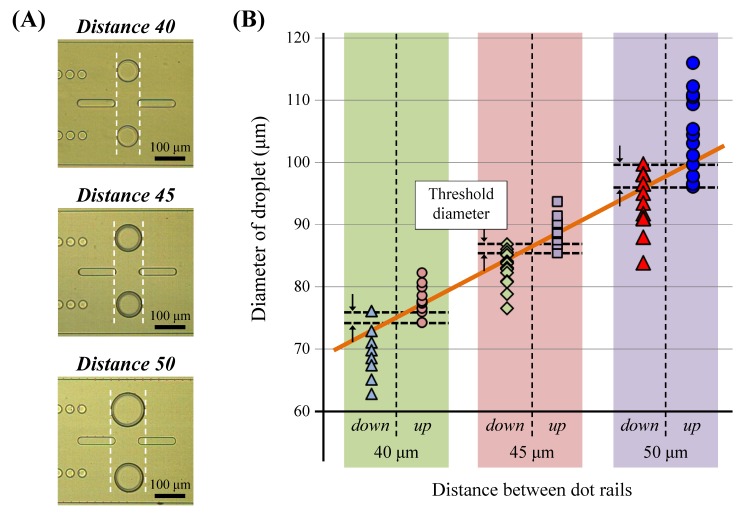
Sorting the results of water droplets using different rail distances. (**A**) Captured images of droplets sorted into different rails by size (images of droplets were treated to evaluate them in the same position). (**B**) Droplet volumes sorted by different rails in each rail type and the threshold diameter of droplet transfer.

The selectively transferred large and small droplets were guided downstream, as shown in Figure 6 (Supplementary Material, Video S3, a case of the 45 μm rail and DI water with mineral oil). The sizes of the droplets were measured in the area without dot structures. The transferrable droplet size changed according to the distance between the two rails.

The threshold diameters of the droplet transferring in the case of the 40, 45, and 50 μm rail-pair distances were approximately 75, 86, and 98 μm, respectively. Furthermore, the widest overlap range of droplet transfer to both rails was smaller than 4 μm. The diameters increased proportionally with the rail-pair distance to almost twice the distance. These results indicate that the outer boundary of droplets should be entered dot rail's area above 10 μm to obtain sufficient Laplace pressure for the position change and that large area is required when the size of target droplet is larger. However, the range was approximately 2 μm which is smaller than the overlap range above, thus the threshold diameter for sorting can be designed simply without the complex consideration of the flow field and droplet behavior.

**Figure 7:**
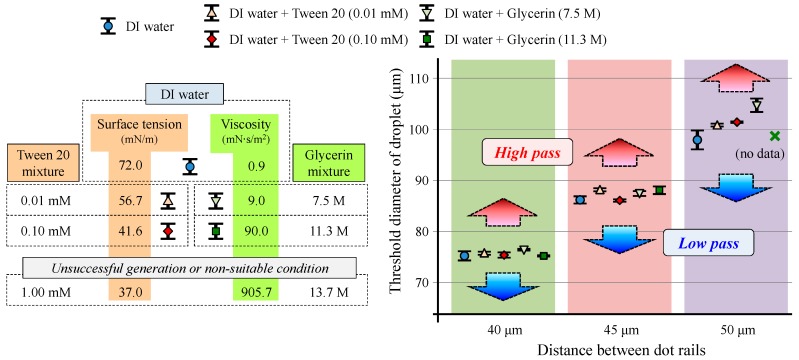
Threshold diameters of sorted droplets using different droplet phase materials by tuning surface tension or viscosity.

Finally, the sorting performance of droplets with different physical properties was evaluated, as shown in Figure 7. Compared to the deionized water, the addition of tween 20 and glycerin decreased the surface tension and increased the viscosity of water, respectively [41,42]. With the exception of some cases of unsuitable flow conditions for the droplet generation, the droplets were sorted successfully by the dot rails. The range of threshold diameter for selective transfer was almost the same as the results using pure water, even though the properties were changed by the additives. There was no significant shift in threshold diameter for the sorting. The overlap ranges were also uniform, whereas the distances between the rails were 40 and 45 μm. The maximum range of threshold diameter using the rail pairs of 40 and 45 μm was smaller than 3 μm for all droplet types. In contrast, the rail-pair distance of 50 μm exhibited more unstable results. The maximum threshold range rose to almost 10 μm. We considered that droplets that were too large influenced the position and outer boundary of the droplet owing to partial deformation such as non-circular deformation. 

The flow rate depends on the droplet material and size but should be limited to utilize the proposed sorting principle. In the aforementioned device design and evaluated conditions, the maximum total flow rate into the device which allowed droplet sorting was 1 μL/min. Because the strong drag force due to high flow speeds neglected the Laplace pressure for droplets in the dot structure, the dot rail can no longer guide the droplet pass. Thus, large dot structures, especially wide dot patterns are necessary to increase the Laplace pressure influencing droplet behavior for the sorting of droplets at high flow speeds. 

Furthermore, even when the droplets were guided by the rail, droplets on the original rail alighted from the rail in the angular area in some cases. This behavior is also caused by the balance of drag force and Laplace pressure because the angle of the dot array requires a position shift of the droplets in the vertical direction and carrier flow pushes the droplets in a parallel direction to the channel.

In cases where the flow rate was high, droplet diameter was large, or the angle of original rail was high, drag force in a parallel direction was too high compared to the lateral force for droplet guiding along the original rail. Thus, the biased force balance prevented the droplets from gaining time to move along the rail and drove them out from the rail. 

In contrast, the close gap between the droplets resulted in unstable droplet guiding in the angular rail area. The flow speed around the droplets was locally high because the droplets played a role of the moving wall. Thus, the increased flow speed around the droplets disturbed the stable guiding of other droplets after sorting. The practical minimum gap between the droplets was approximately double the droplet diameter.

Therefore, the dimensions of the structure and flow conditions should be carefully considered for the stable droplet sorting depending on the target size.

## 5. Conclusions

In this paper, a simple and passive high-resolution droplet sorting method was presented. Rail pairs of dot arrays allowed stable guiding and selective transfer of droplets for size-dependent sample sorting based on the width of the droplet boundary. Since the structure and method utilized the Laplace pressure of droplets and a change in the droplet position without lateral flow for the sorting, the physical properties—except the size—had a minimal influence on the sorting results. 

The threshold diameter of the droplet transfer onto a new rail increased proportionally with the distance between the rail pairs. In contrast, the thresholds were almost uniform even if the properties, particularly viscosity and surface tension, of the droplets were changed. 

The proposed structure is a useful element as a passive droplet filter. When droplet-based mixture has varying properties such as size, density, viscosity, and surface tension, the structure can sort or simply separate the targets by size only. Furthermore, the combinations of line rail developed in our previous research and dot rail can increase the number of sorting categories and improve the sorting precision. 

In addition to integration with other sorting elements, it is expected that the structure can be used as a pre- or post-element in large scale chemical or biological platforms. Since the structure does not require additional flow control and complex manipulation, it can be combined with other conventional functional elements without a complicated design modification. Thus, we believe that the proposed method and structure will provide more improved results in droplet-based research.

## Supplementary Materials

The following are available online at http://www.mdpi.com/2072-666X/9/10/513/s1, Video S1: Transferring of large droplets to the new rail. Video S2: guiding of small droplets along the original rail. Video S3: sorted droplets into different rail ways (in downstream).
